# Anti-Müllerian Hormone Levels in Adolescence in Relation to Long-term Follow-up for Presence of Polycystic Ovary Syndrome

**DOI:** 10.1210/clinem/dgaa949

**Published:** 2020-12-22

**Authors:** Mirte R Caanen, Henrike E Peters, Peter M van de Ven, Anne M F M Jüttner, Joop S E Laven, Marcel H A van Hooff, Cornelis B Lambalk

**Affiliations:** 1 Department of Reproductive Medicine, Amsterdam UMC, Vrije Universiteit, HV Amsterdam, the Netherlands; 2 Clinical Epidemiology and Biostatistics, Amsterdam UMC, Vrije Universiteit, HV Amsterdam, the Netherlands; 3 Division of Reproductive Endocrinology and Infertility, Department of Obstetrics and Gynaecology, Erasmus University Medical Center, CA Rotterdam, The Netherlands; 4 Department of Obstetrics and Gynaecology, Sint Franciscus Hospital, PM Rotterdam, The Netherlands

**Keywords:** polycystic ovary syndrome, anti-Müllerian hormone, adolescence, menstrual cycle irregularities, oligomenorrhea

## Abstract

**Context:**

Anti-Müllerian hormone (AMH) measured in adolescence as biomarker for prediction of adult polycystic ovary syndrome (PCOS) is doubtful but not substantiated.

**Objective:**

To investigate whether serum AMH levels and other PCOS-associated features in adolescence can predict the presence of PCOS in adulthood.

**Design and Setting:**

A long-term follow-up study based on a unique adolescent study on menstrual irregularities performed between 1990 and 1997.

**Participants and interventions:**

AMH was assayed in 271 adolescent girls. Data on PCOS features were combined with AMH levels. In 160 of the 271 (59%) participants, we collected information in adulthood about their menstrual cycle pattern and presence of PCOS (features) by questionnaire 2 decades after the initial study.

**Results:**

AMH was higher in adolescent girls with oligomenorrhea compared with girls with regular cycles, median (interquartile range): 4.6 (3.1-7.5) versus 2.6 (1.7-3.8) μg/L (*P* < 0.001). Women with PCOS in adulthood had a higher median adolescent AMH of 6.0 compared with 2.5 μg/L in the non-PCOS group (*P* < 0.001). AMH at adolescence showed an area under the receiver operating characteristic curve for PCOS in adulthood of 0.78. In adolescent girls with oligomenorrhea the proportion developing PCOS in adulthood was 22.5% (95% CI, 12.4-37.4) against 5.1% (95% CI, 2.1-12.0) in girls with a regular cycle (*P* = 0.005). Given adolescent oligomenorrhea, adding high AMH as factor to predict adult PCOS or adult oligomenorrhea was of no value.

**Conclusions:**

Adolescent AMH either alone or adjuvant to adolescent oligomenorrhea does not contribute as prognostic marker for PCOS in adulthood. Therefore, we do not recommend routine its use in clinical practice.

Anti-Müllerian hormone (AMH) has been identified as an important factor involved in ovarian function. The use of AMH as a marker for ovarian reserve has gained more attention (1-4). AMH is released from ovarian granulosa cells, resulting in serum levels that are proportional to the number of early developing follicles (5). Previous studies have consistently shown that serum AMH levels are (2- to 4-fold) increased in adult women with PCOS (6-9), and therefore indicate a potential relevance in PCOS diagnosis. Limited data are available on normal values of AMH in adolescents (10-13).

Irregular menstrual cycles during puberty are considered to be physiologic. However, oligo- or secondary amenorrhea is, in about half of the cases, persistent into (young) adulthood (14) and is then recognized as symptom of the polycystic ovary syndrome (PCOS), the most common endocrine disorder in women of reproductive age (15-17). The diagnosis of PCOS in adults is well-defined by the Rotterdam criteria: oligo- or anovulation, hyperandrogenism, and/or polycystic ovaries (18).

Defining appropriate diagnostic criteria for diagnosis of PCOS in adolescence remains a challenge (19). Diagnostic issues result first from impropriety of transvaginal ultrasound in patients being virgin and in addition the uncertainty regarding the clinical significance of polycystic ovarian morphology (PCOM) in these youngsters. Also, interpreting clinical and biochemical signs of hyperandrogenism is more difficult in adolescents (20-22). Finally, the most important diagnostic issues result from physiological menstrual irregularities in the postmenarchal period (23-25). Therefore, the use of adolescent AMH levels as a diagnostic marker or as a predictor of PCOS, would be of great value. So far, the use of AMH as biomarker has not been validated in adolescents and, in absence of reliable data, it is advised to not use it as a single test for the diagnosis of PCOS (19).

Despite difficulties in diagnosing PCOS during adolescence, it is important to identify adolescents at risk. They may benefit earlier from lifestyle adaptations on future long-term health by reducing development of metabolic disturbance and cardiovascular risk factors associated with PCOS (26, 27). Awareness and intervention can start during adolescence and more benefit will be achieved regarding women’s health.

Stored serum and data from an extensive adolescent study in the 1990s (24, 25, 28-30) has provided the unique opportunity to study AMH in adolescents and relate these values to the occurrence of PCOS later in life. The aim of the present study is to investigate the relationship between AMH levels and typical PCOS-associated features in adolescence and their value as prognostic marker to diagnose PCOS in adulthood.

## Materials and Methods

### Study Population

#### Pubertal Onset of Menstrual cycle abnormalities: a prospective study

We used data from a unique adolescent study performed between 1990 and 1997 that collected endocrinology and body markers of various menstrual cycle patterns in adolescents in a general population. The was extensively described previously (24, 25, 28-30). In summary, the Puberty Onset Menstrual cycle abnormalities, a Prospective study (POMP) investigated the natural course of pubertal-onset menstrual cycle irregularities. This was done in a combined rural and urban region of the Netherlands and was performed by the division of Reproductive Medicine from the Vrije Universiteit Medical Center, Amsterdam, in close collaboration with the regional Youth Health. The original cohort consisted of 2480 adolescent schoolgirls who were interviewed on their menstrual cycle pattern. A subset of the participants was invited for physical examination, blood sampling for endocrine evaluation, and transabdominal ultrasound.

All girls with oligomenorrhea were invited to participate for follow-up after 3 years. For every oligomenorrheic girl, 2 girls with a regular menstrual cycle were invited as controls.

#### Current study

**Figure 1.**
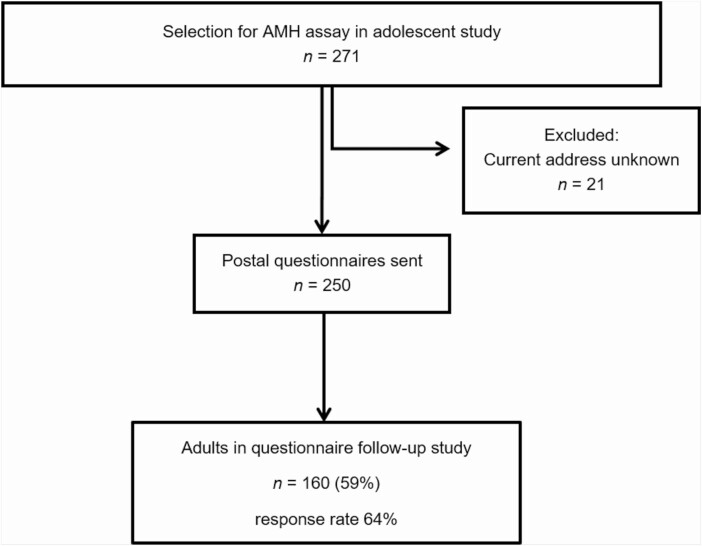
Flow chart of the study population follow-up study.

For the current analysis, we identified the original participants in the POMP study (median age, 15.1 years) who did not use hormonal contraceptives at the time of blood sampling. Available stored serum samples of 271 adolescents were assayed for AMH. Available adolescent data from the original study were combined with AMH levels. We approached these participants in September 2016 to collect information regarding long-term follow-up. Data were obtained by an extensive postal questionnaire, and when the information was not sufficient or consistent, by mail or telephone contact. In 160 of the 271 (59%) adolescents that were assayed for AMH, we were able to obtain information about their current menstrual cycle pattern and presence of PCOS features and PCOS diagnosis in adulthood (Fig. 1).

Both the POMP study and this long-term follow-up study were approved by the institutional review board, and informed consent was obtained from all participants. The adult follow-up study was registered in the Dutch Trial Registry (trial registration number NTR5871).

### Study Design

#### Adolescent parameters

Details on adolescent data collection were extensively described previously (24, 25). From the adolescent data, we categorized the menstrual cycle pattern in either oligomenorrhea (average menstrual cycle longer than 35 days) or regular cycles (average menstrual cycle shorter than 35 days). Gynecological age was calculated by subtracting the age at menarche from the calendar age (24). Physical examination in adolescence consisted of measurement of length, weight, and waist-hip circumference. Hirsutism was defined as a modified Ferriman and Gallwey score ≥8 (31). Acne was defined as a Plewig and Kligman score ≥1 (32).

For endocrine evaluation, blood samples in adolescents with a regular menstrual cycle were taken between the first and the 10th day of the menstrual cycle. In oligomenorrheic girls, blood samples were also taken during the extended follicular phase but at least 21 days before the next period. LH U/L, FSH U/L, androstenedione (ADION) nmol/L, estradiol pmol/L, testosterone nmol/L and dehydroepiandrosterone sulfate (DHEAS) µmol/L were determined by the best available kits that time, described in detail previously (28). For the current study, AMH was measured using an ultrasensitive immunoassay (pico-AMH, AnshLabs, Houston, TX, USA). The intra-assay coefficient of variation was 4.1%, 1.6%, 1.8%, and 2.8% as determined at concentrations of 29 pg/mL, 126 pg/mL, 304 pg/mL, and 656 pg/mL, respectively. Inter-assay coefficient of variation was 2.5%. The pico-AMH assay (AnshLabs) was extensively validated recently and showed high agreement with the more commonly used Gen II ELISA AMH assay (Beckman Coulter) (33).

For the current study, we defined hyperandrogenism in adolescence as the presence of hirsutism and/or biochemical hyperandrogenism (according to cutoff points of the endocrine laboratory of the Vrije Universiteit University Medical Center Amsterdam: testosterone >2 nmol/L and/or DHEAS > 12 µmol/L and/or ADION > 9 nmol/L).

Transabdominal ultrasound of the ovaries was performed in a subset of the study cohort and details are described previously (30). PCOM was defined in the original study as ≥10 antral follicles (2-8 mm) in 1 plane (30, 34) and/or a volume of ≥10 mL in at least 1 ovary.

#### Adult follow-up study: the questionnaire

The questionnaire used in our follow-up study is an adaptation of a well-tested questionnaire used by the Department of Epidemiology of the Netherlands Cancer Institute in a Dutch cohort study on long-term effects of ovarian stimulation on in vitro fertilization (35, 36) and reproductive outcomes in childhood cancer survivors (33). Our questionnaire addressed general health information, menstrual cycle characteristics, self-reported acne, and hirsutism (addressed by a modified Ferriman-Gallwey score assessment).

### Study Outcomes

The respondents who had a menstrual cycle longer than 35 days at the time of the questionnaire were considered to have oligomenorrhea (in case of hormonal contraception use, fertility treatment, pregnancy or breastfeeding, or the menstrual cycle pattern during the year before these events was used).

PCOS in adulthood was defined as having self-reported oligomenorrhea and clinical hyperandrogenism (presence of hirsutism or severe acne) and/or reporting PCOS as medical diagnosis and/or reason of treatment by a physician (37-39)

### Statistical Analysis

All statistical procedures were performed using SPSS version 22.0 (SPSS Inc., Chicago, IL, USA) and STATA 14.1. Data on baseline characteristics, hormonal measurements and ovarian morphology is reported as mean (SD) when normally distributed, median (interquartile range) when not normally distributed, or number (percentages). Normality was assessed visually by means of separate QQ plots for subgroups with regular menstrual cycle and oligomenorrhea. Normally distributed variables were compared using *t* tests, whereas the nonparametric Mann-Whitney test was used when data were not normally distributed. AMH was compared between groups using a 2-sample test after a log-transformation to satisfy the requirement of normally distributed residuals. Strength of association between AMH and clinical signs of hyperandrogenism and hormonal measurements was quantified by means of Pearson correlation or Spearman correlation, where the latter was used for ordinal and skewed data. The prognostic value of AMH at adolescence for PCOS and oligomenorrhea at adulthood was visualized by means of receiver operating characteristic (ROC) with the prognostic value quantified by means of area under the curve (AUC). Measures for diagnostic accuracy were determined using cutoff values for AMH levels used in clinical practice. The original POMP study used a stratified sampling scheme in which adolescents with oligomenorrhea were oversampled. Compared with the general population, in the follow-up study cohort, adolescents with oligomenorrhea were overrepresented and those with regular menstrual cycles underrepresented. Therefore, when making comparisons between subgroups (other than between women with and without oligomenorrhea in adolescence), sampling weights were used to take into account the sampling scheme (28). The same sampling weights were used for reweighing the diagnostic accuracy measures and prevalences to the original sample of adolescent girls which in adulthood participated in this follow-up study.

## Results

### AMH and PCOS Features in Adolescence

#### Baseline characteristics

For this study, there were 271 adolescent girls with a median age of 15.1 years. Eighty girls (29.6%) reported oligomenorrhea. Baseline characteristics of the study population according to menstrual cycle pattern are shown in Table 1. The regular cycling girls were significantly older (calendar age and gynecological age) compared with the girls reporting oligomenorrhea. No significant differences were found for age at menarche, body mass index (BMI), and presence of acne between the menstrual cycle pattern groups. Waist-hip ratio was lower in the regular group compared with the oligomenorrhea group.

**Table 1.** Adolescent baseline characteristics according to menstrualcycle pattern

	Regular (n* = *191)	Oligomenorrhea (n* = *80)	*P* value^*a*^
Age, y	15.1 (14.9-15.6)	15.1 (14.8-15.3)	**0.03**
Gynecological age, y	2.3 (1.4-2.8) (n = 187)	1.8 (1.2-2.4)	**0.02**
Age menarche, y	13.1 (12.5-13.7) (n = 187)	13.3 (12.5-13.9)	0.19
Body mass index, kg/m^2^	20.1 (18.6-21.2) (n = 190)	19.9 (17.9-21.7) (n = 78)	0.65
Waist-hip ratio, cm/cm	1.38 (1.32-1.43) (n =* *165)	1.40 (1.35-1.46) (n = 64)	**0.04**
Acne^*b*^			
Yes	113 (64.6)	48 (67.6)	0.65
No	62 (35.4)	23 (32.4)	
Hyperandrogenism^*c*^			
Yes	31 (16.2)	24 (30.0)	**0.01**
no	160 (83.8)	56 (70.0)	

Data are presented as median (interquartile range) or n (%). Statistically significant (*P* < 0.05) is in bold.

^*a*^Mann-Whitney *U* test for continuous variables, χ ^2^ test for categorical variables.

^*b*^Plewig and Kligman score ≥1.

^*c*^Biochemical and/or modified Ferriman Gallwey score ≥8.

#### Hormonal measurements

Table 2 shows a comparison of the hormone concentrations between the 2 groups according to menstrual cycle pattern in adolescence. Median AMH level was significantly higher in the oligomenorrhea group than in the regular group: 4.6 and 2.6 μg/L. In the subset of 24 girls with oligomenorrhea plus hyperandrogenism, we measured the highest median AMH of 6.8 µg/L. LH, ADION, estradiol, and testosterone were also significantly higher in the oligomenorrhea group than in the regular group.

**Table 2.** Adolescent hormonal measurements according to menstrual cycle pattern

Menstrual cycle pattern	Regular (n = 191)	Oligomenorrhea (n = 80)	*P* value^*a*^
Hormone			
AMH, μg/L	2.6 (1.7-3.8)	4.6 (3.1-7.5)	**<0.001**
LH, U/L	2.7 (1.8-3.8)	4.3 (2.7-6.0)	**<0.001**
FSH, U/L	4.9 (3.9-5.8)	4.7 (3.9-5.5)	0.103
ADION, nmol/L	5.0 (3.8-6.7)	5.7 (4.5-7.7)	**0.005**
Estradiol, pmol/L	115.0 (88.0-142.0)	137.5 (100.0-182.3)	**0.002**
Testosterone, nmol/L	1.0 (0.9-1.3)	1.3 (1.0-1.6)	**0.001**
DHEAS, μmol/L	4.1 (2.8-6.3)	4.8 (3.5-6.2)	0.071

Data are presented as median (interquartile range). Statistically significant (*P* < 0.05) is in bold.

Abbreviations: ADION, androstenedione; AMH, anti-Müllerian hormone; DHEAS, dehydroepiandrosterone sulfate.

^*a*^Mann-Whitney *U* test.

AMH correlated with levels of testosterone (*r* = 0.20, *P* < 0.01), LH (*r* = 0.31, *P* < 0.01), FSH (*r* = -0.15, *P* = 0.02), DHEAS (*r* = 0.12, *P* = 0.06), and ADION (*r* = 0.31, *P* < 0.01), but not with adolescent acne *(r* = 0.021, *P* = 0.74).

#### AMH and ovarian morphology

Original data on ovarian morphology (ovarian pattern and volume) of 151 adolescents could be combined with AMH serum levels. Adolescents with PCOM had overall significantly higher serum AMH levels compared with the non-PCOM group (Table 3). After stratification for menstrual cycle type, AMH levels were found to be significantly higher in the group with PCOM compared with those without PCOM in both subgroups with regular cycle and oligomenorrhea.

**Table 3.** Geometric mean of adolescent serum AMH levels (μg/L) with 95% CI and median with interquartile range

Stratum	PCOM	Non-PCOM	*P* value^*a*^
Regular cycles^*b*^	GM: 3.5 (2.9-4.4)	GM: 2.4 (2.0-3.0)	**0.013**
(n = 95)	Median: 3.3 (2.3-6.3)	Median: 2.6 (1.8-3.8)	
	(n = 36)	(n = 59)	
Oligomenorrhea^*b*^	GM: 5.4 (4.4-6.5)	GM: 3.5 (2.5-4.7)	**0.012**
(n = 56)	Median: 6.0 (3.7-8.4)	Median: 3.6 (2.7-4.4)	
	(n = 38)	(n = 18)	
Combined group	GM: 3.8 (3.2-4.6)	GM: 2.5 (2.1-3.0)	**0.002**
(n = 151)	Median: 3.4 (2.5-6.2)	Median: 2.6 (1.8-3.8)	
	(*n* = 74)	(n = 77)	

Stratified for menstrual cycle pattern and abdominal ultrasound features of the ovaries. Combined group analyses with observations reweighted according to the sampling scheme. Data presented as GM: geometric mean with 95% CI or median with interquartile range. A total of 25 girls with regular menstrual cycles and 18 girls with oligomenorrhea started the use of hormonal contraceptives in the time between blood sampling and ovarian ultrasound. Statistically significant (*P* < 0.05) is in bold.

Abbreviations: AMH, anti-Müllerian hormone; GM, geometric mean.

^*a*^ *t* test on log-transformed AMH level using sampling weights.

^*b*^Adolescent cycle pattern at time of blood sampling.

### PCOS Features in Adulthood

We received a completed questionnaire at adult age reporting on PCOS features in later life from 160 of 271 invited adult participants. The responder group (n = 160) and nonresponder group (n = 111) did not differ in baseline characteristics at adolescent age, (gynecological) age, BMI, presence of hyperandrogenism, and oligomenorrhea (data not shown). The 160 women in the responder group had a median age of 39.6 years at time of follow-up. In this group, 67.5% of the women were classified at adolescent age as regular cycles and 32.5% as oligomenorrhea. Of the adult women, 22.5% (36/160) reported oligomenorrhea and 11.9% (19/160) were identified as having PCOS.

#### Prevalence of adult oligomenorrhea and PCOS in adulthood

After weighting to account for the sampling scheme, 6.9% (95% CI, 3.7-12.5) of the adolescents were estimated to have PCOS at adulthood. In the subgroup with oligomenorrhea at adolescent age, this proportion was estimated to be 22.5% (95% CI, 12.4-37.4). A significantly lower prevalence of 5.1% (95% CI, 2.1-12.0) of the girls with regular cycles during adolescence developed PCOS during adulthood (*P* = 0.005).

Overall, the proportion of women with oligomenorrhea at adulthood was 12.9% (95% CI, 8.2-19.8). This proportion was is estimated to be 37.9% (95% CI, 24.9-53.0) in women with oligomenorrhea at adolescent age and 10.0% (95% CI, 5.4-18.1) in case of regular cycles (*P* < 0.001).

Mean BMI in adulthood was not different in PCOS vs non-PCOS women: 25.7 vs 25.3 kg/m^2^, *P* = 0.82. Also, the mean increase of BMI from adolescence to adulthood did not differ between PCOS women compared with non-PCOS women: 5.35 vs 4.88 kg/m^2^, *P* = 0.78.

#### Predictive value of adolescent AMH

Table 4 shows results for comparison of AMH levels between women with and without PCOS in adulthood, and with and without oligomenorrhea in adulthood (analysis reweighted for sampling scheme). Overall, women with PCOS in adulthood had higher levels of adolescent AMH compared with women without (*P* < 0.001), whereas adolescent AMH levels for women with and without oligomenorrhea in adulthood were found to be borderline different (*P* = 0.074). Comparisons restricted to the subgroup with oligomenorrhea at adolescent age did not reveal differences in AMH levels.

**Table 4.** Serum AMH levels at adolescence according to the presence of oligomenorrhea and PCOS in adulthood

		AMH, µg/L	*P* value^*a*^
A. Total follow-up group, n = 160			
PCOS adulthood	Yes (n = 19)	GM: 4.8 (3.5-6.5)	**<0.001**
		Median: 6.0 (4.4-7.6)	
	No (n = 141)	GM: 2.5 (2.2-2.9)	
		Median: 2.5 (1.7-3.6)	
Oligomenorrhea adulthood	Yes (n = 36)	GM: 3.6 (2.4-5.3)	**0.074**
		Median: 3.9 (2.9-6.9)	
	No (n = 124)	GM: 2.5 (2.2-2.9)	
		Median: 2.4 (1.7-3.6)	
B. Selection of study subjects with oligomenorrhea at adolescent age, n = 52			
PCOS adulthood	Yes (n = 12)	GM: 3.6 (1.8-6.9)	0.54
		Median: 4.4 (1.9-7.6)	
	No (n = 40)	GM: 4.2 (3.5-5.1)	
		Median: 4.2 (3.1-6.2)	
Oligomenorrhea adulthood	Yes (n = 21)	GM: 4.2 (2.9-6.2)	0.81
		Median: 4.4 (3.1-7.6)	
	No (n = 31)	GM: 4.0 (3.2-5.0)	
		Median: 3.9 (2.8-6.1)	

Data presented as GM: geometric mean with 95% confidence interval, or median with interquartile range. Statistically significant (*P* < 0.05) is in bold.

Abbreviations: AMH, anti-Müllerian hormone; GM, geometric mean; PCOS, polycystic ovary syndrome.

^*a*^Mann-Whitney *U* test.

**Figure 2.**
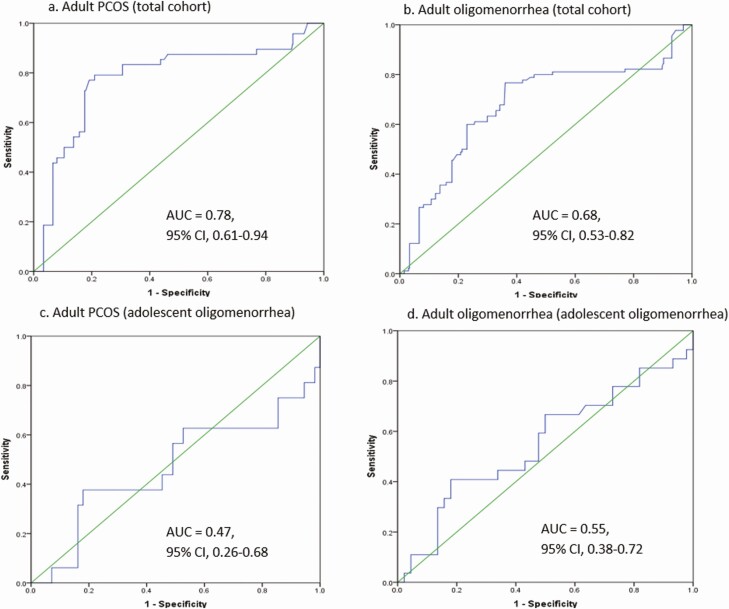
Prognostic value of AMH at adolescence for PCOS and oligomenorrhea in adulthood. (A, B) Total group (n = 160). (C, D) Group with oligomenorrhea in adolescence (n = 52). Abbreviations: AMH, anti-Müllerian hormone; PCOS, polycystic ovarian syndrome.

ROC curve analysis of adolescent AMH levels showed an AUC for PCOS and oligomenorrhea in adulthood of 0.78 and 0.68, respectively (Fig. 2A/B). For girls with oligomenorrhea at adolescence, the AUC was poor (Fig. 2C/D) and AMH as an additional prognostic determinant to oligomenorrhea did not improve the predictive prognostic accuracy. The sensitivity and specificity of serum AMH level at adolescent age was evaluated for PCOS and oligomenorrhea in adulthood, by using cutoff values applied in clinical practice according to the ROC curve. The sensitivity for PCOS in adulthood with a cutoff value for AMH of 6 µg/L was 50.0% and the specificity was 87.0%. This cutoff level for AMH of 6 µg/L resulted in a positive predictive value (PPV) for PCOS of 22.3% and a negative predictive value of 95.9%. Among the girls who presented with oligomenorrhea in adolescence, the PPV (for AMH cutoff, 6 µg/L) was similar, 27.4%. However, the negative predictive value was lower: 79.7% (40).

#### Predictive value of PCOS features at time of adolescence

In various subsets of adolescent girls, the prevalence of PCOS and oligomenorrhea in adulthood was calculated (Tables 5 and 6). These results were reweighted for the sampling scheme. When estimated for the general population, the prevalence of PCOS in adulthood was 6.9%. In girls with oligomenorrhea during adolescence, the prevalence of PCOS in adulthood increased to 22.5%. Combining adolescent oligomenorrhea with the presence of PCOM or a serum AMH level > 6 μg/L resulted in the same prevalence of adult PCOS: 22.2% and 27.4%, respectively. For adolescent girls with oligomenorrhea, increased AMH and an increased BMI (n = 5) resulted in the highest prevalence of PCOS: 71.9%.

**Table 5.** Prevalence (%) of PCOS and oligomenorrhea in adulthood for different subgroups of adolescents

	Various subsets of adolescent girls, with presence of 1 or more PCOS-associated features												
	Total group (n* *= 60)	Oligo (n* *= 52)	PCOM (n = 45)	HA (n = 31)	AMH ↑ (n = 30)	LH ↑ (n = 20)	BMI ↑ (n = 40)	Oligo and PCOM (n = 26)	Oligo and HA (n = 15)	Oligo and AMH↑ (n = 17)	Oligo and PCOM and HA (n = 11)	Oligo and PCOM and HA and AMH ↑ (n = 6)	Oligo and BMI ↑ and AMH ↑ (n = 5)
PCOS in adulthood	6.9 (3.7-12.5)	22.5 (12,4-37.4)	10.9 (3.5-29.5)	8.7^*a*^	22.3 (7.8-49.3)	25.5^*a*^	9.4 (3.0-25.8)	22.2 (8.9-45.4)	10.4 (2.3-36.9)	27.4 (8.7-59.9)	15.2 (3.0–51.3)	16.7 (0.9-81.4)	71.9 (21.9-95.9)
Oligo in adulthood	12.9 (8.2-19.8)	37.9 (24.8-53.0)	14.4 (5.8-31.2)	13.9^*a*^	26.9 (11.1-52.1)	37.1^*a*^	17.5 (7.6-35.5)	36.0 (18.3-58.5)	36.7 (13.6-68.0)	50.1 (23.6-76.6)	30.5 (10.8-61.4)	50 (9.1-90.9)	85.9 (13.1-99.6)

Data are presented with 95% CI.

Abbreviations: AMH ↑ = anti-Müllerian hormone cutoff level > 6 µg/L; BMI **↑ = **body mass index > 75th percentile (21.4); HA, hyperandrogenism; LH ↑ = cutoff level > 6 U/L; Oligo, oligomenorrhea; PCOM, polycystic ovarian morphology on ultrasound; PCOS: polycystic ovary syndrome.

^*a*^95% CI could not be computed because of reweighing a stratum with a single observation only.

**Table 6.** Prevalence (%) for presence of oligomenorrhea and PCOS in adulthood separate for strata defined by categories of adolescent cycle pattern and quartile groups of BMI

Cycle pattern in adolescence:	Adolescent BMI			
	<P25 (BMI < 18.4)	P25-50 (BMI 18.4-20.0)	P50-75 (BMI 20.0-21.4)	>P75 (BMI > 21.4)
Regular cycles	n = 25	n = 26	n = 30	n = 27
PCOS in adulthood	14.5 (4.2-39.7)	1.5 (0.1–10.9)	1.0^*a*^	5.3 (0.6–33.1)
Oligomenorrhea in adulthood	15.2 (4.6-39.9)	9.4 (2.3–31.5)	5.1^*a*^	12.6 (3.4–36.6)
Oligomenorrhea	n = 15	n = 14	n = 10	n = 13
PCOS in adulthood	31.5 (10.4-64.7)	9.0 (65.4-97.8)	0^*a*^	40.1 (13.8-73.5)
Oligomenorrhea in adulthood	47.4 (19.3-77.2)	24.6 (12.3-43.1)	16.5 (3.1-54.7)	55.0 (23.2-83.2)

Data are presented with 95% CI.

Abbreviations: BMI, body mass index; P, percentile; PCOS, polycystic ovary syndrome.

^*a*^95% CI could not be calculated because of reweighing a stratum with a single observation only or lack of cases.

As mentioned, the overall prevalence of oligomenorrhea in adulthood was 12.9% and with oligomenorrhea during adolescence increased to 37.9%, but when combined a serum AMH level > 6 μg/L, it increased to 50.1%.

Tables 6 and 7 show the prevalences of adult oligomenorrhea and PCOS stratified for adolescent BMI and waist-hip-ratio. Girls with a high BMI (>75th percentile) and oligomenorrhea had the highest chance of developing PCOS or oligomenorrhea in adulthood: PPV of 40.1% and 55.0%, respectively. Girls with a high hip-waist ratio (>75th percentile: > 1.44 cm/cm) and oligomenorrhea had the highest chance of developing PCOS or oligomenorrhea in adulthood: PPV of 33.1% and 49.8%, respectively (Table 7).

**Table 7.** Prevalence (%) for presence of oligomenorrhea and PCOS in adulthood separate for strata defined by categories of adolescent cycle pattern and quartile groups of WHR

Cycle pattern in adolescence:	Adolescent BMI			
	<P25 (WHR < 1.34)	P25-50 (WHR 1.34-1.39)	P50-75 (WHR 1.40-1.44)	>P75 (WHR > 1.44)
Regular cycles	n = 30	n = 27	n = 22	n = 27
PCOS in adulthood	4.3^*a*^	7.1	1.7	8.6 (0.9-49.9)
Oligomenorrhea in adulthood	5.4^*a*^	21.8	2.5	10.9 (1.7-46.9)
Oligomenorrhoea	n = 9	n = 10	n = 12	n = 13
PCOS in adulthood	8.2 (0.7-54.1)	0.0^*a*^	17.7 (2.7-62.0)	33.1 (11.0-66.5)
Oligomenorrhea in adulthood	32.9 (13.8-59.8)	15.2 (3.0-50.9)	23.5 (5.1-63.7)	49.8 (19.6-80.2)

Data are presented with 95% CI.

Abbreviations: BMI, body mass index; P, percentile; PCOS, polycystic ovary syndrome; WHR, waist-to-hip ratio.

^*a*^95% CI could not be calculated because of reweighing a stratum with a single observation only or lack of cases.

## Discussion

We tested the hypothesis that serum AMH level in adolescence could be a helpful prognostic marker for adult PCOS; therefore, we investigated its relationship with the common PCOS-associated features in adolescence and presence of PCOS in adulthood. We clearly show that AMH relates with all PCOS features in adolescence. Women with self-reported PCOS in adulthood had higher levels of AMH as adolescent. However, the potential role of AMH in adolescence as a valuable prognostic marker on its own or in addition for PCOS during adulthood could not be confirmed.

The present study demonstrates that each of the individual PCOS features found in adolescence contribute only to a limited extent in predicting whether PCOS developed in adulthood. Although the overall prevalence of adult PCOS was about 7%, it increased 3-fold in girls with adolescent oligomenorrhea, whereas in adolescents with regular cycles, it was only 5.1%.

As a single diagnostic marker for PCOS, increased AMH in adulthood showed a PPV for PCOS similar as oligomenorrhea. It is of particular clinical interest to identify among adolescents that present with oligomenorrhea those that who will develop PCOS. Our data substantiate that the addition of AMH levels on top of having adolescent oligomenorrhea do unfortunately not contribute to the further identification of those that will have PCOS as adult. As such, it should not be used in the diagnostic workup of the adolescent with oligomenorrhea. On the other hand, we confirm that BMI in adolescence is a good marker for the persistence of oligomenorrhea into adulthood (25). The adolescent with oligomenorrhea, a high AMH, and a high BMI has a very high chance to develop PCOS or oligomenorrhea later in life. According to our results, PCOM and hyperandrogenism in adolescence showed a relatively low positive predictive value and proved not to be of particular value in predicting PCOS during later life.

So, given adolescent oligomenorrhea, none of the single or a combination of classical PCOS features was associated with a significant better prediction for PCOS later in life. Why does elevated AMH, more or less acknowledged as diagnostic criterion in adult women, not contribute to identification of future PCOS among adolescents? First, elevated AMH is not definitively considered as unique PCOS feature (41). Second, strong fluctuations in AMH, particular in young women are reported (42), which may potentially lead to intrinsic inaccuracy. Finally, the possibility exists that we misclassified PCOS diagnosis in adult life. A considerable number of the adult women with oligomenorrhea were not classified as having PCOS, which is in accordance with the literature (43, 44). PCOS diagnosis in our study was made by 2 of 3 Rotterdam criteria (information about ovarian morphology was not available to us) or by reporting PCOS as a medical diagnosis. Not unlikely a number of women is underdiagnosed (45) because they have regular cycles or despite the oligomenorrhea they did not seek medical treatment because naturally concepted pregnancies may have occurred.

Despite the limited additional prognostic value of AMH in adolescence, we confirm that AMH levels are higher in girls with PCOS associated features. Our data are in line with most but not all previous studies in adolescents (10, 13, 46) showing that AMH is significantly higher in adolescents with oligomenorrhea compared with girls with regular cycles. Also, in line with previous studies in adolescents and adults (9, 13, 47, 48), our data show that AMH was significantly correlated with hyperandrogenism.

AMH is proposed as a substitute for antral follicle count in diagnosing PCOS in adults (1, 49, 50). In line with previous adolescent studies (11, 51, 52), the present study demonstrates that adolescent PCOM is associated with elevated AMH concentrations. Regarding ovarian ultrasound appearance, AMH positively correlates with ovarian volume and the number of follicles in the ovaries determined by ultrasound. The ultrasound criteria for the definition of polycystic ovaries may be less applicable in adolescents because it is known that many adolescents have enlarged, multifollicular ovaries (53, 54). Given that, nowadays, ultrasound criteria for PCOM are determined by vaginal ultrasound, especially in adolescents, replacing ultrasound for AMH measurement would be strongly desirable, because of the impropriety of transvaginal ultrasound in virginal patients.

To our knowledge, this is the first long-term longitudinal follow-up study investigating AMH and other PCOS features in adolescence as a prognostic marker for PCOS in adulthood. We were able to investigate AMH in a large group of adolescents that were not on hormonal contraceptives, and to parallel this with PCOS-related features. Although the data collected in this study are valuable, they do have their limitations. A first limitation is that the PCOS diagnosis in adulthood was based on a self-reported questionnaire. We used a validated questionnaire and previous studies that used self-reported PCOS yielded highly acceptable rates of accurate identification (37-39, 55-57). Second, it would be of interest to perform a multiple logistic regression analysis to identify factors associated with PCOS at adulthood. Unfortunately, the sample size of this study (n = 19) is too small to perform such a multivariable analysis. We therefore had to limit ourselves to a model with AMH only corrected for oligomenorrhea at adolescence. Third, the hormonal samples were taken years ago and stored before the measurement of AMH became available. But of note, the comprehensive adolescent data were gained by a physician during a hospital visits and AMH was measured using an acknowledged modern laboratory method and measured AMH values are comparable with existing literature. Finally, the finding of biologically plausible differences between the various adolescent supports contemporary their validity.

In conclusion, this study confirms and extends that AMH measured in adolescence has the potential to distinguish between PCOS features: menstrual cycle irregularities, hyperandrogenism, and polycystic ovarian morphology. However, most important, is that AMH as an adjuvant diagnostic marker in adolescents with menstrual cycle irregularities does not contribute to the diagnosis of frank PCOS later in life.

## Data Availability

The datasets generated during and/or analyzed during the current study are not publicly available but are available from the corresponding author on reasonable request.

## Abbreviations

ADION: androstenedione
AMH: anti-Müllerian hormone
AUC: area under the curve
BMI: body mass index
DHEAS: dehydroepiandrosterone sulfate
PCOM: polycystic ovarian morphology
PCOS: polycystic ovary syndrome
POMP: Puberty Onset Menstrual cycle abnormalities, a Prospective study
PPV: positive predictive value
ROC: receiver operating characteristic

## Additional Information

***Disclosures:*** The authors have nothing to disclose.
